# Restoration of urban agriculture soil by autochthonous fungal biodiversity

**DOI:** 10.1007/s00253-026-13877-z

**Published:** 2026-05-28

**Authors:** Matteo Florio Furno, Michela Tramontini, Elisa Gaggero, Monica Rigoletto, Debora Fabbri, Mery Malandrino, Paola Calza, Giovanna Cristina Varese, Federica Spina

**Affiliations:** 1 Dept. of Life Sciences and Systems Biology, University of Turin, Viale Pier Andrea Mattioli 25, 10125 Turin, Italy; 2 Dept. of Chemistry, University of Turin, Viale Pietro Giuria 7, 10125 Turin, Italy

**Keywords:** Urban agriculture, Fungi, PAHs, Bioaugmentation, Biosurfactants

## Abstract

**Abstract:**

The persistence of PAHs in soil poses a significant challenge for common strategies of soil recovery, affecting, for instance, the general perception of urban agriculture, being highly exposed to different sources of pollution. Sustainable strategies for their mitigation are needed, such as mycoremediation, since it relies on fungi to transform pollutants into products with lower environmental burden. The current study investigates fungi of polluted soil, aimed at urban agriculture, with the intent of creating an active microbial consortium capable of transforming the pollutants of interest and decreasing the toxicity of the site. The analysis of key community members revealed a diverse mycobiota, mainly dominated by Ascomycota. Among the 28 isolated taxa and 16 genera, the most abundant ones were *Aspergillus*, *Scedosporium*, *Trichoderma*, and *Fusarium*. Fungal isolates were evaluated for their potential to grow in the presence of LMW and HMW PAHs, showing a significant difference due to inter- and intra-specific variabilities. Data showed that some fungi can also produce biosurfactants. Coupling this information with the capability of fungi to grow on carriers, six fungi were selected for in situ soil remediation. Biostimulation and bioaugmentation (with strains in co-cultures) were evaluated for pollutant mitigation. The best results were obtained by a microbial consortium that also revealed high tolerance to HMW PAHs. The chronic ecotoxicity of the soil did not show any effect on *Eisenia fetida*, with the only exception of biostimulation, which led to a 44% toxicity decrease. This study provides a foundation for fungal-based approaches for soil bioremediation and quality recovery, unveiling for the first time the capability of many species to inhabit PAHs-contaminated soil and be actively involved in their degradation.

**Key points:**

• *The microbiota of polluted soil was mainly dominated by Ascomycota.*

• *The isolated 28 taxa mostly belong to Aspergillus, Scedosporium, Trichoderma, and Fusarium.*

• *Many fungi degrade PAHs and/or produce biosurfactants.*

• *A mix of millet and canary seeds worked as an efficient fungal carrier.*

• *Biostimulation and bioaugmentation were tested against a PAHs-polluted soil.*

**Supplementary Information:**

The online version contains supplementary material available at 10.1007/s00253-026-13877-z.

## Introduction

Human activities have changed during the past decades, which has led to a multifunctional use of our landscapes. The number of people located in metropolitan cities is constantly growing (Lal 2020) and urban areas are conquering territories at the expense of the rural surroundings. Industrialization has led to the extension of the cemented areas, changing the natural landscape into an anthropic environment with a net impact on humans and the entire ecosystems (Zhu et al. 2011). Changes in land cover and use both contribute to terrestrial biodiversity loss. The United Nations (UN) estimates that one fifth of the Earth’s land area is degraded, causing alarming effects to biodiversity (United Nations 2020). Hence, the preservation of terrestrial ecosystems while exploiting their natural resources needs to become a leading topic, since loss of biodiversity and pressures on ecosystem services are becoming an actual global environmental risk.

Several strategies can be implemented, including the restoration of marginal urban areas. Indeed, in agreement with EU objectives of eliminating land consumption by 2050, several municipal administrations have shown a growing interest in restoring urban and peripheral green areas by assigning their management to associations or to private citizens for the cultivation of flowers, fruits, and vegetables (Appolloni et al. 2021). In this context, urban agriculture is becoming a worldwide phenomenon, capable of attracting 100–200 million urban farmers worldwide (Orsini et al. 2013). The protection of the territory, the reduction of land consumption and hydrogeological risk are all achievable goals, while having only a moderate pressure on our ecosystem. In Italy, community gardens have recently increased diffusively. Turin has highly promoted this phenomenon with 2 million m^2^ dedicated to vegetable gardens and agricultural areas.

However, being located within or close to an anthropized environment, community gardens are widely exposed to pollution due to various anthropogenic sources such as industry, vehicular traffic, energy production and waste treatment plants which increase the concentration of organic and inorganic contaminants in the atmosphere (Li et al. 2018). As a result of atmospheric depositions, pollutants accumulate in urban soils and plants growing therein, having direct access to the trophic chain and ultimately impacting human health. Acknowledging this risk can also lead to the altered perception of the urban agriculture benefits by potential stakeholders, who may identify products as low quality (Di Fiore et al. 2021). However, this should not slow down those movements devoted to the valorisation of marginal areas. It is instead becoming essential to understand the risk and the chemical scenario the community gardens are exposed to. In particular, polycyclic aromatic hydrocarbons (PAHs) are the pollutants mainly responsible for these toxic effects, sadly known to be carcinogenic or mutagenic (Bamforth and Singleton 2005).

The microbial community populating these sites is reacting to these external stressors, but with low transformation rates. Indeed, the natural degradation of PAHs is slow, causing the continuous accumulation of PAHs in the environment that ultimately become persistent in soil (Morillo et al. 2007). Wherever PAHs concentration exceeds the threshold limits, decontamination strategies should be implemented. Many remediation techniques based on chemical-physical approaches are currently available, such as solvent extraction, chemical oxidation, photocatalytic degradation or electrokinetic techniques (Gan et al. 2009). However, many of these processes are inherently expensive, disruptive, and not eco-friendly. Moreover, in an urban horticulture context, the conservation of soil fertility is essential, and the unique balance of chemical, physical and biological components must also be protected through remediation strategies that do not introduce new possible contaminants into the environment (Yi and Sung 2015). The need to ensure these prerogatives has increased the interest in biological techniques that use living organisms capable of degrading contaminants into less toxic products: biostimulation and bioaugmentation can reduce the pollution, while having a low environmental impact (Aso and Obuekwe 2024). Biostimulation involves the addition of nutrients or substrates to the polluted soil in order to stimulate the activities of autochthonous microbes (Kuppusamy et al. 2016), while bioaugmentation is aimed at introducing or increasing a microbial population with degradative capabilities (Das and Chandran 2011). Even though both autochthonous and allochthonous microorganisms can be used, the former ones are already adapted to the specific pollutants present in the soil of interest and may show peculiar metabolic skills (Spini et al. 2018).

Talking about microorganisms of soil, fungi are surely the main organisms involved, being responsible for the carbon cycle. Moreover, they can adapt to anthropic extreme environments, where they can explore the soil thanks to their *habitus filamentous* while triggering their unspecific metabolism mediated by non-ligninolytic and ligninolytic enzymes (Bamforth and Singleton 2005). This powerful enzymatic arsenal includes numerous extracellular enzymes (i.e., laccases, peroxidases, cytochrome P450) (Al-Zaban et al. 2021; Aranda et al. 2017; Janusz et al. 2017; Yu et al. 2024).

Several reports explored the transformation skills of fungi against PAHs (Omoni et al. 2024). Previous studies indicated that fungi belonging to the genera *Peniophora*, *Phlebia*, *Pleurotus*, *Penicillium*, and *Cladosporium* are responsible for degrading PAHs in the soil and aquatic environments (Aranda et al. 2017; Birolli et al. 2018; Lee et al. 2014; Pozdnyakova et al. 2010). In past years, many researches employed fungi coming from different isolation sources (Lee et al. 2014; Pozdnyakova et al. 2010), and the identification of fungi adapted to PAHs-contaminated environments is not a common practice. Despite the lack of dedicated research, this strategy may trigger the success rate of bioremediation, hence the living mycobiota of this polluted ecological niche may include strong strains adapted not only to the complex carbon source (e.g., pollutants) but also to the peculiar soil conditions.

In most cases, native fungal isolates were used to degrade individual PAHs in model conditions (Aranda et al. 2017; Lee et al. 2014; Simanjorang and Subowo 2018) but rarely these fungal-based approaches have been challenged in a more complex scenario. Bench-scale results can only show the potential of fungi, but how they would behave in polluted soil or water has to be verified. Currently, information about decontamination of a polluted soil by a microbial-based solution at a large scale is still scarce.

The present study makes instead the bioremediation of a contaminated area (Turin, Italy) as its final and primary goal. The hazard removal of the polluted soil was the main goal, being the site otherwise designated for urban agriculture. Biostimulation was compared with a bioaugmentation process, based on selected autochthonous fungi directly isolated from the polluted site. Technical parameters affecting the performances of the *in-field* application were also investigated, such as the proper system to vehiculate the microbial consortium into the polluted soil by using adequate lignocellulosic carriers.

## Materials and methods

### Reagents

Acetone (≥ 99.8%) has been provided from VWR International Srl. Hexane (≥ 99%), anthracene D-10 (> 98%), PAHs Calibration Mix (TraceCERT, 10 µg/mL in acetonitrile) and the other reagents have been provided from Merck, Sigma Aldrich. Compost Florawiva® has been provided by Acea Pinerolese (Pinerolo, TO, Italy) whose composition is reported in Table S1.

### Polluted site characterization

The site of interest is located in the urban area of Turin (Campana Street garden, CA; GPS coordinates, 45° 3′ 14″ N, 7° 40′ 57″ E; Turin, Italy), dedicated to urban agriculture. Soil samples were collected using a core drill at 40-cm depth. Before sampling, 15-mm topsoil was removed to avoid collecting the turf (Gaggero et al. 2020).

Soil samples were quartered, air dried for 5 days, sieved on a stainless-steel sieve with 2-mm mesh, ground in a centrifugal ball mill, stored in polycarbonate jars, and analyzed to determine PAHs concentration. Samples were extracted with a microwave system, based on EPA 3546 (U.S. Environmental Protection Agency (EPA) 1993). The obtained solutions were spiked with 50-ppb anthracene D-10 as internal standard, and then purified using Florisil SPE cartridges (Supelco®, Merck) previously conditioned with 4 mL hexane. The purified extracts were concentrated to a final volume of 1.5 mL with a rotavapor (Büchi Italy, Cornaredo Italy).

GC–MS analysis was conducted using an Agilent 8860 GC/5977 MSD equipped with a 7693 A autosampler, and a HP-5MS UI column (30 m × 0.25 mm with a 0.25-µm-thick film). All the injections were performed using a splitless mode and the injector was maintained at 280 °C. Helium was used as carrier gas with a column flow rate of 1.5 mL/min and an electron impact ionization was employed.

The column temperature was initially maintained at 60 °C for 0.5 min and programmed to heat up to 325 °C at a rate of 15 °C/min. The mass spectrometer was operated in SIM mode to analyze US EPA’s sixteen PAHs priority pollutants (Table S2).

Calibration curves were carried out using standard solutions prepared by diluting concentrated stock solutions (PAHs calibration mix). Limits of Detection (LOD) and Limits of Quantification (LOQ) are shown in Table S3.

### Fungal isolation

To isolate the fungal community adapted to the contaminated soil, liquid enrichment was set using a minimal medium where aromatic and aliphatic hydrocarbons represent the sole carbon source. The compounds were chosen due to their abundance in the target soil (Table S4), namely phenanthrene (Phe), pyrene (Pyr), perylene (Pe) and eicosane (Ei). Stock solutions were prepared in ethanol 95% for Pyr and Phe, in chloroform for Pe and in methyl-t-butyl ether for Ei. Paraffin oil (Pa) and a crude mineral oil (Oi) were chosen as a representative mix of alkanes and PAHs.

Ten g of soil were added to a 250 mL glass flask with 90 mL sterile minimal medium (MM) and a target analyte: Phe, Pyr, Pe 200 ppm; paraffin oil and crude oil 1% v/v. The MM was a modified Czapek medium: NaNO_3_ 2 g/L, K_2_HPO_4_ 1 g/L, NH_4_Cl 1 g/L, mineral solution (MS) 10 mL, trace metal solution (TMS) 1 mL, and antibiotics (2 mL/L gentamicin, 444 µL/L (piperacillin/tazobactam). MS (100 mL): KCl 5 g, MgSO_4_ 7H_2_O 5 g, FeSO_4_ 7H_2_O 0.1 g. TMS (100 mL): ZnSO_4_ 7H_2_O 1 g, CuSO_4_ 5H_2_O 0.5 g.

Bottles were incubated in the dark at 25 °C at 120 rpm. After 7 days, 5 mL of the supernatant was transferred to another flask with fresh MM and the corresponding target analyte. Three consecutive subcultures were done.

After 1 month, the liquid was inoculated in agar plates (MM with contaminants); serial dilutions were prepared. Plates were incubated in the dark at 25 °C and regularly controlled. Forming colonies were isolated in pure culture in a rich medium (MEA: 20 g/L glucose, 20 g/L malt extract, 2 g/L peptone, 18 g/L agar).

### Fungal identification

Fungal isolates were identified by means of a polyphasic approach. Fungi were firstly morphologically classified based on specific taxonomical keys (Samson et al. 2014). Molecular analyses were performed by sequencing specific genomic regions, as described by Spina and collaborators (2021). In detail, genomic DNA of each strain was extracted from about 100 mg of mycelium using the NucleoSpin® Plant II kit (Macherey–Nagel), according to the manufacturer’s instructions. The quality and quantity of extracted DNA were spectrophotometrically measured by using Infinite M200 (TECAN Trading, Austria). DNAs were stored at −20 °C for further studies. Amplification of specific genic regions (e.g., ITS, beta-tubulin, actin) occurred in a T100™ thermal cycler (Bio-Rad). PCR products were subjected to electrophoresis in a 1.5% agarose gel in Tris–Borate-EDTA (TBE) buffer for 20 min at 80 mV. Amplification products were detected by staining the gel with ethidium bromide and displayed under UV light in a Gel Doc™ XR system (Bio-Rad). Taxonomic assignments were inferred by querying consensus sequences against the nucleotide database of NCBI (GenBank). Fungal isolates of the same species were subjected to dereplication analysis (microsatellite M-13 as a primer), in order to identify unique strains (Poli et al. 2016). Fungal strains are preserved at the *Mycotheca Universitatis Taurinensis*, the Fungal Collection of the University of Torino. Sequences have been deposited in NCBI, from MZ497084 to MZ497099.

### Fungal characterization

#### PAHs degrader screening

Fungi were inoculated in 96-well microplates, containing a MM with three target pollutants: Pyr and Phe (at 200 ppm), and Pa (1% v/v), as representative of organic and aliphatic compounds. Microplates were incubated at 24 °C in the dark for 3 weeks. Absorbance at 750 nm was periodically spectrophotometrically measured (Infinite M2000, TECAN). Biotic (with glucose) and abiotic controls were carried out. Six replicates were carried out. Fungal growth in the presence of PAHs was compared with the biotic control and expressed as growth percentage.

### Biosurfactant screening

The fungal capability to produce biosurfactants was investigated. Fungi were inoculated in a modified MM (KH_2_PO_4_ 0.3 g/L, MgSO_4_ 0.3 g/L, NaNO_3_ 3 g/L, yeast extract 2 g/L, soybean oil 40 mL/L and glucose 5 g/L) to stimulate their surfactants-producing metabolism. The flasks were incubated at 25 °C and 120 rpm. After 1 week, the culture broth was separated from the mycelium by centrifugation (7,000 × g, 30 min, 4 °C).

The presence of biosurfactant in the supernatant was evaluated by means of the Oil Displacement Area (ODA) as described by Morikawa and collaborators (2000). The ODA test provides information about wetting activities of a sample with surfactant activity: the larger the diameter of the clearance halo, the greater the activity.

### In situ treatment scale-up

#### Carrier colonization

Three lignocellulosic byproducts were used as microbial carriers: a mix of millet and canary seeds (MC, 1:1 w/w), wheat seeds (W), corn and rice husk (CR, 1:2 w/w). The carrier colonization was performed as described by Zanellati and collaborators (2021). Fungi were pre-grown in submerged fermentation with malt extract broth (glucose 20 g/L, malt extract 20 g/L, peptone 2 g/L). Flasks were incubated at 25 °C at 120 rpm. After 7 days, the mycelium was filtered and inoculated into plates with 25 g of sterilized MC, W and CR. The byproducts were moistened at their optimal water content (2:1 w/v) to sustain microbial growth. The final fungal inoculum/biomass ratio was 1:20 w/w. Three replicates were set up. Plates were incubated in the dark at 25 °C for 14 days. The growth rate and colonization rate of the plates were assessed by checking the mycelial mats development around the carrier particles.

#### In situ treatment

Different bioremediation strategies were adopted, based on natural attenuation, biostimulation and bioaugmentation. The bioremediation trials were performed in mesocosms (500 kg each), as shown in Table S5. The soil was mixed with the other supplements (e.g., organic amendment and/or fungi) using a concrete mixer in order to have a homogeneous matrix. Mesocosms were set up in closed vessels (0.8 m^3^) made of high-density polyethylene (HDPE).

Four scenarios were studied:

i) Natural attenuation (control soil, C) was used to compare the efficiency of the proposed treatments.
ii) Biostimulation (BS) was achieved proving an organic amendment (1:10 w/w) (Florawiva, FW, ACEA Pinorolese Industriale S.p.A.) to soil samples, as an additional source of nutrients to boost the metabolism of the microbial community of the soil itself.
iii)  For bioaugmentation (BA) experiments, mesocosms were inoculated with six fungal strains (1:10 w/w). Inoculum preparation started by the establishment of the mycelium of each strain on the selected carrier (MC) as previously described. The inoculum starter (e.g., 2 kg of colonized MC) was used as a starter of the final inoculum (e.g., 20 kg of colonized MC)

iv) The combination of biostimulation and bioaugmentation (BS + BA) was also assessed.

Mesocosms were located in an area of interest and exposed to the occurring weather conditions. Using a core drill, different soil samples were taken at the beginning and after 6 months. Each sample was stored at 4 °C before analyses. Three replicates were prepared for each sample and analytes concentrations were calculated as previously described (see “Polluted sites characterization” section). Quantification was made evaluating the results of the internal standard signal (Table S6). Degradation percentages were calculated comparing PAHs content of each trial with the non-inoculated control (C) to consider only the effects of the different treatments on PAHs biodegradation.

### Ecotoxicological test

The ecotoxicological analyses were carried out at the beginning and at the end of the mesocosm. The test organism, *Eisenia foetida (*earthworm) was selected as it has been widely used as a model organism in soil chronic toxicity assessment. The test was performed according to the standard ISO 11268:1998 (E)-(U.RP.M999) (Spurgeon et al. 2003). The soil (500 g dw) was moistened with deionized water up to 60% of the maximum water holding capacity, and the moisture was maintained during the experiment. For each trail, ten earthworms were inoculated. The soil was incubated at 20 °C with a 16-h photoperiod. Mortality was determined after 4 weeks. Earthworms were then removed, while samples were monitored for 4 weeks. The number of offspring at the end of the experiment was measured.

### Statistical analyses

Data were analyzed by a non-parametric test of Mann–Whitney analysis with SPSS Statistics software version 25 (SPSS for Windows, Chicago, IL, USA) considering statistically significant difference for those with *p*-value ≤ 0.05.

## Results

### Fungal community of the polluted site

The fungal community of a polluted soil, previously dedicated to urban agriculture, was studied. Sixty-two fungal entities, ascribable to 28 taxa and 16 genera were found (Table S7). The isolates were dominated by Ascomycota, with the sole exception of the Basidiomycota *Hyphodermella rosae* MUT 6527. The most frequently isolated genera were *Aspergillus* (17 strains), *Scedosporium* (15 strains), *Trichoderma*, and *Fusarium* (5 strains).

Most of the isolated taxa showed an isolation-substrate specificity: 19 out of 28 species were retrievable only on one pollutant (Table S7). Some fungi were isolated both on alkanes (Ei, five taxa; Pa, two taxa) and aromatic (Pe, four taxa; Py, two taxa) compounds. No exclusive species were found on Phe. Interestingly, Oi showed the highest number of univocal isolated species (6 taxa), demonstrating once again the high adaptation these fungi have been subjected to. Indeed, because of the complex structure of the crude oil, the capability to grow on it clearly denoted unique and powerful metabolic skills.

Some fungi showed a more aspecific growing pathway, whose isolation was not exclusive of a single pollutant (Table 1). Only *Scedosporium dehogii* solely grew in the presence of aromatic compounds, while the other taxa developed regardless of the presence of aromatic and aliphatic compounds as sole carbon source.

**Table 1:** Fungal taxa growing on different substrates: paraffin oil (Pa), eicosane (Ei), phenanthrene (Phe), pyrene (Pyr), perylene (Pe), and crude oil (Oi)

Taxa	Pa	Ei	Ph	Py	Pe	Oi
*Aspergillus fumigatus*	x	x			x	x
*Aspergillus nidulans*					x	x
*Aspergillus terreus*	x	x			x	x
*Galactomyces pseudocandidum*		x				x
*Purpureucillium liliacinum*		x			x	x
*Scedosporium apiospermum*		x			x	x
*Scedosporium dehogii*				x	x	x
*Talaromyces trachyspermus*			x			x
*Trichoderma asperellum*			x		x	

Fungal taxa are listed in alphabetical order

Unfortunately, among members of the mycobiota, emerging opportunistic human and animal pathogens (e.g., *A. fumigatus* and *Scedosporium* spp.) were also isolated. Because of safety concerns for bioremediation operators and community garden users, 13 out of 62 strains belonging to species classified as potentially harmful to humans and animals (e.g., H2 and keratinolytic species) were excluded from further analyses. Only harmless strains were used for subsequent trials.

### Pollutant transformation and biosurfactant production

As shown in Table 2, some fungi could use PAHs as sole C source. With only very few exceptions where growth was not observed at all, fungi did exhibit the capability to develop in such extreme conditions. The growth yield was however strongly affected by the presence of the pollutants, with inhibition up to 82%. Overall, phenanthrene seemed to greatly affect the growth in comparison to Pyr, even though the reaction was mostly strain dependent. For instance, *Aspergillus nidulans* MUT 6528 grew more in the presence of Pyr (68.7 ± 5.5%) than with Phe (25.7 ± 1.0%), but many fungi showed the opposite behavior, as *Pichia manshurica* MUT 6551 (Phe 76.4 ± 2.3% vs Pyr 14.6 ± 0.7%). A common trait cannot be drawn among fungi belonging to the same species, which indeed behaved differently once exposed to the pollutants (e.g., *Metarhizium robertsii*).

**Table 2:** Fungal growth (expressed in growth percentage in comparison with the control) and biosurfactants activity at the end of the experiment. When the growth was statistically comparable or even higher than the control, the values are in bold emphases. Biosurfactant production is expressed as: 9-cm halo (+ + +), 4- to 5-cm halo (+ +), barely notable halo ( +), absent halo (-). Statistical differences of the growth of each single strain among the pollutants are expressed in superscript lowercase letters

Strains	Phe	Pyr	Pa	BS	Strains	Phe	Pyr	Pa	BS
*A. nidulans*	55.4^a^	57.2^a^	**96.8**^b^	+	***H. rosae* MUT 6527**	76.5^a^	79.7^a^	44.4^c^	+ + +
*A. nidulans* MUT 6528	25.7^a^	68.5^b^	**100.1**^c^	+	***L. canina* MUT 6549**	2.0^a^	41.9^b^	**152.5**^c^	+
*A. niveus* MUT 6535	24.9^a^	36.1^b^	71.1^c^	+ +	***M. robertsii***	77.7^a^	0.9^b^	37.8^c^	+
*A. terreus* MUT 6525	80.1^a^	76.5^a^	71.5^a^	+ +	***M. robertsii* MUT 6526**	26.3^a^	71.3^b^	**117.4**^c^	+ +
*A. terreus* sp.1	65.1^a^	74.0 ^b^	**138.1**^c^	+	***P. camponoti* MUT 6548**	44.7^a^	61.4^b^	**96.4**^b^	+
*A. terreus* sp.2	69.7^a^	49.2 ^b^	**102.7**^c^	+	***Penicillium* sp.* 1***	57.7^a^	65.5^a^	**100.5**^c^	+
*A. terreus* sp.3	55.3^a^	59.2^a^	**94.3**^b^	+ +	***Penicillium* sp.* 2***	36.7^a^	24.4^b^	88.4^c^	+ + +
*A. terreus* sp.4	72.5^a^	46.0^b^	**102.0**^c^	+	***P. manshurica* MUT 6543**	53.2^a^	33.9^b^	**115.1**^c^	+ +
*A. terreus* sp.5	77.8^a^	47.1^b^	**106.2**^c^	+ +	***P. manshurica* MUT 6550**	58.8^a^	60.4^a^	**92.2**^b^	-
*C. pseudocladosporioides* MUT 6536	18.1^a^	41.3^b^	77.6^c^	+	***P. manshurica* MUT 6551**	76.4^a^	14.6^b^	**102.5**^c^	-
*C. rosea*	63.5^a^	18.1^b^	**100.6**^c^	+	***P. lilacinum* MUT 6545**	48.6^a^	33.9^b^	**85.7**^c^	+
*C. rosea* MUT 6529	45.2^a^	**116.5**^c^	**108.2**^c^	+	***P. lilacinum***	48.8^a^	55.4^a^	**88.2**^b^	-
*F. oxysporum* MUT 6537	61.7^a^	**88.7** ^c^	**132.4**^c^	+	***T. trachyspermus* MUT 6542**	51.5^a^	61.1^b^	**89.3**^c^	+ +
*F. oxisporum*	**68.3**^a^	0.4^b^	**78.1**^c^	+ +	***T. crustaceus* MUT 6541**	**90.5**^a^	**98.9**^b^	**115.6**^c^	+
*F. proliferatum* MUT 6544	49.5^a^	**80.9**^b^	**130.3**^c^	+ +	***T. asperellum* MUT 6524**	**78.7**^a^	**130.3**^b^	**158.2**^b^	+ +
*F. solani*	36.2^a^	39.3^a^	**104.4**^b^	+ + +	***T. asperellum* sp.1**	55.6^a^	55.3^a^	**95.4**^b^	+ +
*F. solani* MUT 6538	74.2^a^	59.9^b^	**130.6**^c^	+ + +	***T. asperellum* sp.2**	29.6^a^	28.4^a^	**147.2**^b^	+ + +
*G. pseudocandidum* MUT 6546	34.3^a^	33.5^a^	**112.4**^b^	+	***T. gamsii* MUT 6540**	14.7^a^	61.1^b^	**98.0**^c^	+ + +
*G. pseudocandidum* MUT 6547	41.5^a^	0.5^b^	**110.9**^c^	+	***T. hamatum* MUT 6539**	33.8^a^	37.4^a^	**98.2**^b^	+ + +

Coming from the same polluted soil, it was not surprising to find some strains that were not negatively impacted by the pollutants. In some cases, indeed, the growth with PAHs was comparable (or even higher) with controls where fungi were using a much more accessible source of C, such as glucose. As regards Phe and Pyr, this was true for three (*Fusarium oxysporum*, *Termoascus crustaceus*, *Trichoderma asperellum*) and five (*Clonostrachy rosea*, *Fusarium oxysporum*, *Fusarium proliferatum*, *Termoascus crustaceus*, *Trichoderma asperellum*) strains, respectively. Regarding alkane, this was a common trait for most of the fungi (85%).

Noteworthy, because of a chemically heterogeneous isolation environment, many fungi showed a non-specific growth profile, being active against more than a single pollutant, both aliphatic and aromatic. *T. asperellum* MUT 6524, *T. crustaceus* MUT 6541, *C. rosea* MUT 6529, *F. oxysporum* (MUT 6537 and sp. 1), and *F. proliferatum* MUT 6524 were, by far, the most tolerant isolated fungi, being capable of growing both in the presence of aromatic and aliphatic hydrocarbons. In particular, *T. asperellum* MUT 6524 and *T. crustaceus* MUT 6541 were the only strains capable of exploiting all the target pollutants as well as or even better than the control.

Results of the ODA test are reported in Table 2. Among the 38 fungal strains, broth cultures of seven isolates (18%) showed positive results in the oil-spreading assay and could spread the crude oil immediately upon contact, producing a sharp and wide clarification halo (+ + +). This pattern thereby indicated the presence of compounds with biosurfactant activity. Other 18 strains (46%) led to a smaller but consistent halo (+ +). The remaining fungal cultures did not result in a positive test. The most performing fungi belonged to four genera: *Trichoderma*, *Fusarium*, *Penicillium* and *Hypodermella*.

### Carrier colonization

Strains were evaluated for their capability to transform the pollutants (with particular attention to those that showed an aspecific profile) and for the capability to produce biosurfactants. The 15 most performing strains were used to evaluate their capability to grow on carriers, as the first step for their integration in soil remediation strategy. According to the results shown in Table 3, carriers affected the fungal development. MC was massively colonized by most fungi: 73% (11 out of 15) completely colonized it after only 7 days. Similar results were obtained with W but only by ten fungi (67%). On the other hand, CR was less suitable to sustain the fungal growth: only 53% of fungi (8 out of 15) showed rapid and massive colonization. Three strains (*L. canina* MUT 6549, *P. manshurica* MUT 6543, and *T. crustaceus* MUT 6541) were completely unable to colonize any carrier, ultimately preventing the possibility of applying them for in situ trials despite the good results obtained before. According to the results, MC was selected as a carrier to be used for the in situ treatment.

**Table 3:** Growth of fungi in the presence of the carriers (MC, W, RC), expressed as: homogenous and massive growth (+ +), scattered growth (+), and absent colonization (-)

Strain	MC	W	CR
*Aspergillus nidulans MUT 6528*	+ +	+ +	+
*Aspergillus terreus MUT 6525*	+ +	+ +	+ +
*Aspergillus terreus* sp. *1*	+ +	+ +	+ +
*Aspergillus terreus* sp. *4*	+ +	+ +	+ +
*Aspergillus terreus* sp. *5*	+ +	+	-
*Clonostachys rosea MUT 6529*	+ +	+ +	+ +
*Hyphodermella rosae MUT 6527*	+ +	+ +	+
*Lecythophora canina MUT 6549*	-	-	-
*Metarhizium robertsii*	+ +	+ +	+ +
*Metarhizium robertsii MUT 6526*	+ +	+ +	+ +
*Penicillium* sp.* 1*	+	+	-
*Pichia manshurica MUT 6543*	-	-	-
*Thermoascus crustaceus MUT 6541*	-	-	-
*Trichoderma asperellum MUT 6524*	+ +	+ +	+ +
*Trichoderma asperellum* sp.* 2*	+ +	+ +	+ +

### In situ treatment

The soil was collected directly from the site, exposed to various contamination sources typical of a metropolitan area, which may explain the detection of several hydrocarbons (Table S4). The period of analysis was set at 6 months, since as already suggested by other authors, shorter times may not be sufficient for a complete evaluation of the effectiveness of the treatment, not only in terms of PAHs transformation but also enhancement of soil quality and toxicity reduction (Silva et al. 2022). Taking into account the previous results, the microbial consortium was made of *H. rosae* MUT 6527, *A. terreus* MUT 6525, *T. asperellum* MUT 652*4*, *M. robertsii* MUT 6526, *C. rosea* MUT 6529, *A. nidulans* MUT 6528. For the selection, particular attention was given to their capability to grow on MC, as a technological prerequisite for *in-field* application.

**Fig. 1:**
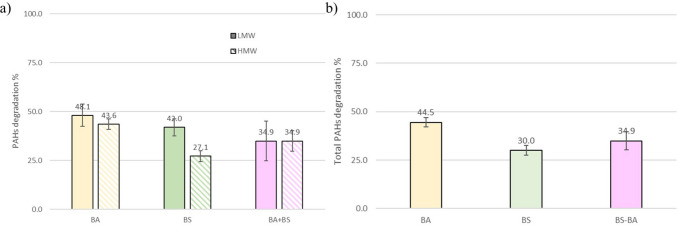
Degradation percentage of the three treatments (BA, BS, BA-BS) of **a)** the total PAHs and **b)** the LMW (black bars) and HMW (gray bars) PAHs

Concerning PAHs quantifications, the comparison of inoculated mesocosms with the non-inoculated control enabled determining the effect of the different treatments rather than the approach of doing nothing and letting the soil rest (Table S8). A significant decrease of total PAHs was observed in all the conditions ranging from 30 to 44.5% of degradation (Fig. 1a; Table S9). In particular, BA, BS, and BA + BS resulted in total PAHs removal of approximately 44.5%, 30.0%, and 34.9%, respectively. Figure 1b shows the transformation percentage of the entire LMW and HMW PAHs fraction. The capability observed before by selected fungi to degrade PAHs was here reflected in thein situ treatments. The highest removal was obtained toward LMW PAHs, in particular when applying BA (48%, 999.5 µg/kg vs 518.8 µg/kg).

**Fig. 2:**
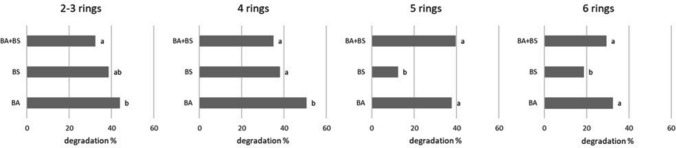
PAHs degradations in mesocosms after 6 months of treatment (BA, BS, BA-BS). Different letters above the bars indicate statistically significant differences between groups according to the Mann–Whitney test (*p* ≤ 0.05)

Bioaugmentation (both BA and BA-BS) showed a wide response against the target pollutants, as the transformation of LMW and HMW PAHs was comparable (48–44% for BA and 35–35% for BA-BS). On the contrary, BS was more efficient to remove LMW PAHs than HMW ones. The natural community alone is stimulated by the presence of organic amendment, but the microbiota is more static and led the degradation of only LMW PAHs, while more complex pollutants remain an issue (27%, 4223.0 µg/kg vs 3076.4 µg/kg). The removal rates of PAHs by BA were 1.1-fold, 1.3-fold, 3.1-fold and 1.7-fold for 2–3-ring, 4-ring, 5-ring, and 6-ring PAHs, compared to BS, respectively (Fig. 2).

This result is surely spiking as it is well known that the recalcitrance of PAHs to biodegradation is increasing with the number of aromatic cycles and their molecular weight. This can be observed in Fig. 2, where the drop of efficiency with the increase of pollutant complexity is more evident in BS (12% and 19% of degradation of 5-ring and 6-ring PAHs, respectively). The other treatments (BA and BA-BS) saw a decrease in process yields but less pronounced (degradation above 30%) and only for 6-ring PAHs. Fungal inoculum confirmed to be crucial for establishing the presence in the soil of a microbial community that facilitates a successful and comprehensive bioremediation.

Acenaphthene, fluorene, phenanthrene, anthracene, fluoranthene, benzo(a)anthracene, chrysene, benzo(b)fluoranthene, benzo(k)fluoranthene, benzo(a)pyrene and pyrene were the most accessible to microbial attack as their concentration was halved in at least one treatment. While for acenaphthene, volatilization can have occurred (Borràs et al. 2010), some compounds are known to be particularly recalcitrant to degradation due to their low bioavailability (e.g., chrysene, benzo(a)anthracene) (Sayara et al. 2011).

**Fig. 3:**
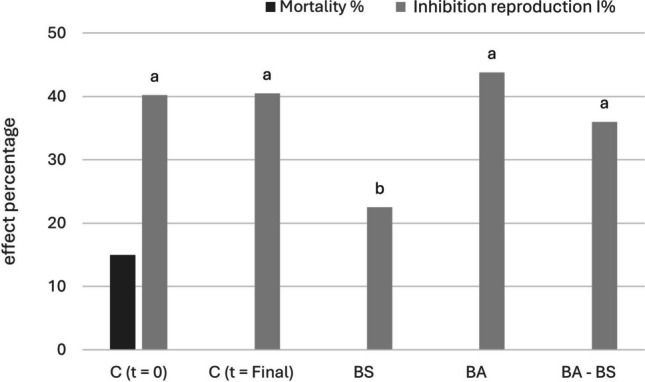
Acute toxicity results (expressed as mortality percentage) and chronic toxicity results (expressed as reproductive inhibition, I%) of the control soil (C) and of the treated soil (BS, BA and BA BS). The black bar indicates the mortality percentage, while the grey bars refer to reproductive inhibition percentage. Different lowercase letters above the bars indicate statistically significant differences between groups according to the Mann–Whitney test (*p* ≤ 0.05)

The results of acute and chronic toxicity tests are shown in Fig. 3. Although the soil originally showed a moderate toxicity (15 ± 5% mortality), no *E. foetida* mortality was observed with any treated soil, indicating a significant reduction of acute toxicity during time. As the chronic toxicity evaluates the toxic effects caused by prolonged exposure, this data is more reliable of the quality of soil during a longer period. No significant differences were detected among samples, with the only exception of BS (44 + 5% toxicity reduction in comparison with control).

## Discussion

### Polluted soil fungal strain isolation

Biodiversity offers an immense basin of environment-friendly options for transformation of contaminants into less harmful non-hazardous compounds. Despite being mostly focused on a few species, the bioremediation of PAHs through fungal-centered solutions is front-edge research, where many fungi have displayed the ability to metabolize PAHs (Haritash and Kaushik 2009). What represents instead a more imminent challenge is their validation against polluted soil. Moving in this direction, studying the diversity of indigenous microorganisms represents a powerful microbial basin to draw from, being capable of degrading different pollutants but also resisting the biotic and abiotic stressors of the polluted site itself. Ultimately, they will result in more resistance to long exposures to contaminants compared to allochthonous microorganisms.

As reported in Table S4, in the selected site, contamination exceeded the Italian threshold limits (D. Lgs 152/2006), posing an optimal case study to investigate the adapted microorganisms, namely that specific subpopulation able to tolerate and metabolize the pollutants of interest. A wide fungal biodiversity was indeed isolated, showing the predominance of Ascomycota. Mucoromycota were instead absent and Basidiomycota represented just a marginal fraction of the isolated population. Lignin degraders basidiomycetes are indeed rarely found in this anthropized ecosystem while they are more typical of wood-decaying conditions (Balami et al. 2021). On the other hand, the predominance of Ascomycota in contaminated soil has been frequently observed (Aranda et al. 2017; Crittenden et al. 2025; Yuan et al. 2018a).

Among the isolated strains, the strong abundance of *Aspergillus*, *Scedosporium*, *Trichoderma* and *Fusarium* did not come as a surprise. Indeed, species belonging to these genera have been often found predominant in polluted soils, showing indeed strong tolerance to high pollutants concentration and intriguing ability to metabolize xenobiotic compounds, such as PAHs (Mineki et al. 2015; Spini et al. 2018).

As expected, several known species of PAH degraders were isolated, including *Aspergillus fumigatus* (Dai et al. 2024), *Aspergillus nidulans* (Khandelwal et al. 2024), *Aspergillus terreus* (Reyes-César e al. 2014), *Fusarium oxysporum* and *Clonostachys rosea* (Omoni et al. 2023), *Fusarium solani* (Fayeulle et al. 2019), *Trichoderma asperellum* (Zafra et al. 2014) and *Trichoderma hamatum* (Zafra et al. 2015). Sixteen species have been reported here for the first time in soil polluted by PAHs: *Aspergillus niveus*, *Cladosporium pseudocladosporioides*, *Fusarium proliferatum*, *Galactomyces pseudocandidum*, *H. rosae*, *Lecythophora canina*, *Metarhizium robertsii*, *Neurospora sitophila*, *Penicillium camponoti*, *Pichia manshurica*, *Purpureocillium lilacinum*, *Scedosporium apiospermum*, *Scedosporium aurantiacum*, *Talaromyces trachyspermus*, *Thermoascus crustaceus*, and *Trichoderma gamsii.* In many cases, data is missing about these specific species but information of closely related species in polluted soil is available (Obi et al. 2020; Reyes-César et al. 2014). For five genera (*Hyphodermella*, *Lecythophora*, *Metarhizium*, *Pichia*, *Purpureocillium*), this study represents instead the first report of their capability to populate significantly polluted soil.

Although most fungi were isolated from a single pollutant, nine strains stood up, being isolated in the presence of multiple ones (Table 1). Noteworthy, among these species, fungi belonging to the genera *Aspergillus*, *Trichoderma*, *Purpureocillium*, and *Talaromyces* have been already reported to colonize polluted areas (Benguenab and Chibani 2020; Mineki et al. 2015; Shanahan et al. 2015) and use several PAHs or an *n*-alkane as a sole carbon source, such as Phe and Pyr and Ei (Hadibarata and Tachibana 2009; Zafra et al. 2015).

The presence in the polluted soil of emerging opportunistic human and animal pathogens (e.g., *A. fumigatus* and *Scedosporium* spp.) cannot be considered outliers, as adaptation skills developed in polluted environments may fortuitously predispose fungi toward human opportunistic pathogenesis. These fungi can be considered ubiquitous in many terrestrial environments, with *Scedosporium* spp. populating soil, compost and plant debris of temperate climates (Rougeron et al. 2018). Many studies revealed that many PAHs-degrading strains are closely related to human-pathogenic fungal species (Prenafeta-Boldu et al. 2005). Indeed, many studies confirmed the capability of potential pathogen fungi to degrade pollutants. Thanks to their various enzymatic pathways that include many lignin-modifying enzymes (Poirier et al. 2023), *Scedosporium* strains are effective against aliphatic and aromatic compounds (Prenafeta-Boldu et al. 2005; Yuan et al. 2018b). Despite this general background, they can cause a wide range of infections mostly on immunocompromised patients. The risk–benefit balance leans heavily toward a more conservative management, excluding them from being re-inoculated in the soil where they could get into contact with at-risk society.

### Fungal strain selection for in-field applications

Thirty-eight fungal strains were selected aiming to identify the most promising ones for *in-field* application, in terms of efficiency and competitiveness in a polluted environment. Indeed, bioremediation agents need peculiar skills which are not limited to the efficient degradation of PAHs. They will first need to compete with autochthonous organisms without weakening the ability to reduce pollution. Moreover, being hydrophobic substances, PAHs could be hardly accessible to microorganisms due to poor mass transfer caused by strong or irreversible sorption on the soil matrix (Samanta et al. 2002). This chemical barrier cannot be broken without solubilizing agents, i.e., biosurfactants, whose production can be done by the adapted metabolism of the microbial population of a polluted site. *In-field* bioremediation is often weaker compared to laboratory tests, mainly due to the poor bioavailability of aromatic and aliphatic hydrocarbons. Therefore, for the success of bioremediation, selected fungi should be capable of overcoming this problem by secreting effective surfactants.

First, it was important to verify the adaptation skills of the isolated fungi, by addressing their capability to grow in the presence of target pollutants. With such a various and complex pollutant scenario, it is important to understand whether fungi can tolerate and grow in their presence. Many fungi have shown to be particularly active against low-molecular-weight compounds (LMW, ≤ three rings) such as naphthalene and phenanthrene, while few strains could degrade high-molecular-weight PAHs (HMW, ≥ four rings), which instead are more toxic and remain almost unchanged (Moghimi et al. 2017). Here the presented results sound surprising, since only 11 strains (29%) did have a marked preference of Phe as growth substrate (Table 2). The other fungi grew better with Pyr (36.8%) or showed no difference at all (34.2%). On the contrary, several studies highlighted how LMW PAHs are more susceptible to microbial degradation than HMW PAHs (van Elsas et al. 2019; Ghosal et al. 2016). Among 150 fungi belonging to Ascomycota, Basidiomycota and Mucoromycota, 59% of them were tolerant to Phe and only 30% to Pyr (da Silva et al. 2003). As often happens in the literature, sometimes different outcomes can be found. For instance, degradation rate of *Peniophora incarnata* was higher for HMW than LMW pollutants (Lee et al. 2014). Zafra and collaborators (2015) showed high degradation skills by *Trichoderma* spp. against LMW PAHs (e.g., Phe), but even better yields against HMW (up to 63% for Pyr and 80% for benzo[a]pyrene) in strongly polluted soils. In the present study, it can be assumed that since the chemical profile of the polluted soil included both LMW and HMW PAHs, autochthonous microbiota responded by evolving various and often aspecific metabolic pathways.

Interestingly, some fungi were not affected at all by the presence of the pollutants, highlighting their strong evolution skills that allowed them to grow comparably with glucose. While for PAHs, this was a matter of a few species (*Clonostrachy rosea*, *Fusarium oxysporum*, *Fusarium proliferatum*, *Termoascus crustaceus*, *Trichoderma asperellum*), this was instead a common behavior for fungi growing with Pa. As regards fungi belonging to *Aspergillus*, *Penicillium* and *Fusarium* genera, many reports have already demonstrated their capability to degrade alkane. For instance, several species of *Aspergillus* and *Penicillium* showed good degradation skills against *n*-alkanes C13–C18 (Elshafie et al. 2007). *Aspergillus terreus*, *Penicillium* spp., *Trichoderma harzianum* and *F. solani*, isolated on polluted soils, degraded aliphatic hydrocarbons (Colombo et al. 1996). Data of *A. terreus* sp. 1, *F. oxysporum* MUT 6537, *F. proliferatum* MUT 6544, *F. solani* MUT 6538, *L. canina* MUT 6549, *T. asperellum* MUT 6524 and sp. 2 should be underlined, as Pa stimulated a higher (above 30%) growth than the control. In some cases, this cannot be considered a novelty, as their activity against aliphatic hydrocarbons has been already reported as well as their presence in highly contaminated soil (Colombo et al. 1996; Husaini et al. 2008; Spini et al. 2018). *Fusarium proliferatum* and *L. canina* have never been associated with aliphatic hydrocarbon pollution before.

Many fungi were very versatile, being able to tolerate both aliphatic and aromatic compounds, intriguing skills for future performances against real multicomponent polluted soil. *T. asperellum* MUT 6524, *T. crustaceus* MUT 6541, *C. rosea* MUT 6529, *F. oxysporum* (MUT 6537 and sp. 1), and *F. proliferatum* MUT 6524 reached the same growth with aromatic and aliphatic hydrocarbons and with glucose. Particular attention should be given to *T. asperellum* MUT 6524 and *T. crustaceus* MUT 6541 because they showed a versatile and aspecific pattern against all the tested pollutants. To the best of the authors’ knowledge, this is the first time that *T. crustaceus* has been efficiently associated with PAHs degradation. Previous reports only targeted PCBs (Mouhamadou et al. 2013). Creosote (a mixture of PAHs, phenolic and heterocyclic organic compounds) degradation was determined for another species of the same genus, e.g., *Thermoascus auriantiacus* (Ghaly et al. 2011). Several *Trichoderma* species were associated with the ability to metabolize a variety of both LMW and HMW PAHs, even in the presence of heavy metals (Atagana 2009; Verdin et al. 2004)*.* The study of Zafra and collaborators (2015) reported that several *Trichoderma* species exhibited high tolerance to PAHs, making them of particular interest for bioremediation purposes. *T. asperellum* showed the highest tolerance level (6,000 mg L^−1^) of a mixture of Phe, Pyr, and benzo[a]pyrene. Alkane degradation by *Trichoderma* spp. is not a surprise. *Trichoderma* sp. transformed alkanes both in liquid fermentation (77%) and in soil (40%) (Hadibarata et al. 2007). However, this is the first report of *T. asperellum* capability to act against alkanes.

The heterogeneity in reported data worldwide can be surely affected by strain variability phenomena that cannot be underestimated. This study is not an exception but instead remarks, once again, the importance of selecting the correct strain, rather than limiting to species evaluation. Indeed, among the 38 strains tested, more than one strain was screened for nine species. Growth on pollutants clearly showed intraspecific traits; strains never behaved similarly. In extreme cases, the growth was even absent with one pollutant for one strain while the other seemed to be barely affected by its toxicity. Just as an example, the two strains of *M. robertsii* did not display the same growth profile: while MUT 6526 grew with Pyr (71.3%) and Pa (117.4%), the other strain used only Phe (77.7%) being incapable of handling Pyr (0.9%) and Pa (37.8%).

To address the challenges of PAHs, the capability of transforming this compound is surely a primary requisite. However, there is a growing interest in comprehensive strategies for accelerating the entire degradation process. Working with organic pollutants, their bioavailability cannot be underestimated, nor those phenomena that help their sequestration in soil. Biosurfactant-producing microorganisms show promise in this regard, enhancing the accessibility of PAHs to the microbial populations. If applied in a bioaugmentation approach, fungi can produce biosurfactants directly in situ, increasing the solubility of PAHs that become easily available to the degrading strains.

Fungi were then screened for biosurfactant production using oil displacement techniques, being correlated to the concentration of biosurfactant in solution (Morikawa et al. 2000). More in detail, ODA can detect biosurfactant production in low levels, and measuring the diameter of the clear zone provides information about the concentration of biosurfactant production (Al-Hawash et al. 2019), even though having a complex matrix and possibly different biosurfactant classes, the correct interpretation of data may be difficult. Biosurfactants production was less consistent among the tested strains with only seven of them, belonging to the genera *Trichoderma*, *Fusarium*, *Penicillium*, and *Hypodermella*, producing a clearing halo. *Fusarium*, *Penicillium*, *Aspergillus* and *Trichoderma* spp. are known to be good biosurfactant producers (Piegza et al. 2021; Sanches et al. 2021; Sánchez 2024; Spina et al. 2018)*.* On the other hand, to the best of the authors’ knowledge, this is the first time that the production of biosurfactants by the basidiomycetes *H. rosae* has been reported. These fungi aroused interesting follow-up as fungal biosurfactants are less commonly reported in the literature compared to bacterial ones (Andrade et al. 2024). Further studies should be performed to identify and characterize the biosurfactant produced by each fungus.

### Fungi immobilization

In the present study, carriers were chosen to respect these paradigms. MC, W and CR are environmentally friendly, cheap, and easily accessible materials. The choice of the most efficient carrier is a critical step, and not every material is suitable for microbial immobilization (Dzionek et al. 2016). The use of immobilized microorganisms has been demonstrated to strengthen their efficiency in bioremediation processes (Angelim et al. 2013; Wang et al. 2007). Immobilization of fungi first can protect them from the shear force, and those varying environmental conditions (e.g., pH and temperature) that could occur during starter preparation, storage and soil inoculum. In this protected form, fungi can also better tolerate other stressors posed by the presence of toxic PAHs (Omoni et al. 2024). While limiting their mobility (at least in the first stage of the soil treatment), carriers may ultimately help the viability and the catalytic activity of fungi in the polluted environment (Dzionek et al. 2016). In soil pollution, ligninocellulose-based materials are often preferable, being environmentally friendly, since no recovery is needed after input into the system. In literature, several natural organic carriers (such as corncob, plant fibers, husk of sunflower seeds) have been tested, demonstrating their effectiveness in bioremediation processes (Carabajal et al. 2016; Cubitto and Gentili 2015). Offering a protective niche, fungi can better compete with the autochthonous microbiota and, thanks to alternative sources of nutrients (e.g., cellulose, hemicellulose, proteins, etc.), they can activate a strong metabolism against the pollutants. In the present study, the three carriers interact differently with the fungi, and only MC allows a wide and strong fungal development, posing the basis for its use into further steps. Testing the capability of fungi to grow on carriers is a technological go-no-go step that cannot be underestimated. Indeed, three strains including some of the best-performing ones in previous tests (e.g., *P. manshurica* MUT 6543 and *T. crustaceus* MUT 6541), did not grow at all, ultimately influencing their use as bioremediation agents. They surely showed great skills to degrade the pollutants of interest at lab scale, but they probably would have issues to confirm this in real environments due to the impossibility of performing strong and homogeneous inoculum.

### Considerations on efficiency of tested bioremediation strategies

The strategies adopted by nature itself to overcome a polluted environment should inspire researchers in the field. Microorganisms usually co-exist and interact with each other: in the presence of xenobiotics, two or more microbial populations of different genera and species take over, acting together as a complex system. To restore contaminated soil, the use of a single microorganism could be highly risky; microbial consortia can serve instead unknown functions potentially inducing alternative degradation pathways and metabolites. They can be equipped with broad enzymatic capacities entailed to enhance the rate and extent of biodegradation, due to diversities of metabolic pathways allowing an efficient transfer of products and metabolites from one strain to the other, up to the complete transformation to water and carbon dioxide, or to unharmful metabolites (Li et al. 2021). A microbial consortium is then robust, effective, and adaptable to varying conditions. These features represent a clear physiological and metabolic advantage with respect to a single organism, whose performances may be low and restricted (Qian et al. 2020). Microbial consortia have been successfully used to convert lignocellulose (Puentes-Téllez and Falcao Salles 2018), industrial waste (Jaspal et al. 2021), volatile organic compounds (Lv et al. 2025), pesticides (Saghee and Bidlan 2018), plastic polymers (Qi et al. 2021), etc.

It is important to formulate a microbial consortium able to guarantee the biotransformation of the various pollutants present on the site and compete for space and nutrients with the autochthonous microflora. Moreover, microbial consortia are more robust to environmental variations and can better survive nutrient limitation thanks to metabolites exchanging or by trading molecular signals (Chaudhary et al. 2017). In the present study, the microorganisms were therefore selected taking into account eco-physiological complementarities and a functional redundancy. Previous experiments helped to select the most active fungi against the pollutants of interest as well as those that could help the consortium efficiency by increasing the accessibility of pollutants themselves. Moreover, only those strains capable of colonizing carriers were considered since they do not arouse any technological constraint to *in-field* application. Therefore, only a few isolates were considered potential candidates for a quantitative in situ assessment of their capacity to degrade PAHs: *H. rosae* MUT 6527, *A. terreus* MUT 6525, *T. asperellum* MUT 652*4*, *M. robertsii* MUT 6526, *C. rosea* MUT 6529, *A. nidulans* MUT 6528. Some of these species have been previously assessed for hydrocarbon, but in most of the cases literature data are limited to a couple of reports (Table 4).

**Table 4:** Information about previous studies of the fungal species used in the microbial consortium

Strain	Contamination of isolation source	Hydrocarbon	Metabolic pathway involved	
*Aspergillus nidulans*	Metal scrapping facility	Spent engine oil, naphthalene, fluorene, phenanathrene, anthracene, pyrene	-	Hock et al. (2018); Khandelwal et al. (2024)
*Aspergillus terreus*	PHAs-contaminated soil, crude oil–contaminated soil	Pyrene, benzo(a)pyrene, phenanathrene	Hydroxylation by cytochrome P-450 monooxygenase followed by conjugation with sulfate ion	Capotorti et al. (2004); Reyes-César et al. (2014)
*Clonostachys rosea*	-	phenanathrene	-	Omoni et al. (2023)
*Hyphodermella rosae*	-	-	-	-
*Metarhizium robertsii*	-	*n*-hexadecane, *n*-octacosane, phenanthrene	Hydroxylation by cytochrome P450 monooxygenases	Huarte-Bonnet et al. (2018)
*Trichoderma asperellum*	Crude oil–contaminated soils	Phenanthrene, pyrene, benzo[a]pyrene	Increase in the activity of catechol 1,2- and 2,3-dioxygenases	Zafra et al. (2014, 2015)

This consortium includes PAHs degraders and biosurfactants producers, as they all have the capability to develop into carriers and then be properly vehiculated in the polluted soil. The concept of introducing biosurfactants into polluted soil is not new, but the common approach is to add free biosurfactants such as rhamnolipids (Ghorbannezhad et al. 2021). Their vehiculation through the entire section of the site as well as their stability in time can both represent an issue. The here proposed strategy would overcome these limitations; hence the mycelial network may colonize the soil and produce over time biosurfactants when necessary. Bioaugmentation can then be a long-lasting process.

As regards the bioaugmented trials, this data cannot clearly state whether the effect is due solely to the strains themselves or to their interaction with the endogenous microbial community. When bioaugmentation was involved, data were significantly different from those obtained with biostimulation only. Otherwise, the competition of the inoculated fungi with the native microorganisms could deeply limit their operativity (Zafra et al. 2014). In the present study, the origin of the consortium offered a physiological advantage, ultimately favoring its permanence against contaminated soil.

Fungi were indeed found capable to compete with the autochthonous microbiota, a skill not to be underestimated since it is a primary cause of weak bio-based remediation process. For instance, fungal activity was higher treating sterile soil, and it was strongly inhibited by the addition of the two soil bacteria (Borràs et al. 2010). Phenanthrene, anthracene, pyrene, acenaphthene and fluoranthene were almost completely removed (above 80% degradation) by *Phanerochaete chrysosporium*, but the process yields dropped (38.94–62.89%) in unsterile soil (Bishnoi and Kumar 2008). The choice to inoculate fungi isolated from the polluted soil was a successful strategy, hence not all fungi are equivalent. Fungi were indeed actively degrading PAHs, while this was not the case in other studies. The addition of *Trametes versicolor* did not enhance the remediation process of a PAHs-contaminated soil (Sayara et al. 2011), but it should be underlined that this fungus was collected from a microbial collection, and did not have any relation with polluted environments.

The natural community was active against the target pollutants, even though at a lower extent than BA (Figs. 1 and 2). This was more evident for complex hydrocarbons, such as 5- and 6-rings. Comparing BA and BA-BS data, it can be observed that degradation levels are slightly affected by the supplementation of organic amendment, mostly for LMW PAHs. This observation came as a surprise, as literature often reports the advantages of adding nutrients and, more specifically, organic waste amendment to stimulate microbial activity (Omoni et al. 2024). Mancera-López and collaborators (2008) reported that PAHs removal from different fungal strains in a silty-loam soil polluted with a complex mixture of petroleum hydrocarbons is increased from two to sixteen times with the biostimulation treatment with nutrient solution. This is not a unique case study, as contrasting evidence has been also reported. The degradation obtained by adding *Talaromyces helicus* to a polluted soil was completely inhibited by the addition of nitrogen and phosphorus sources (Fayeulle et al. 2019). Similarly, Lladó and collaborators (2013) saw that the addition of lignocellulosic amendment promoted both growth and activity of native soil populations but, in the presence of white rot fungi inoculation, a marked antagonistic effect occurred. It cannot therefore be excluded that the use of the basidiomycetes *H. rosae* MUT 6527 in the fungal consortium may have caused the effect observed in this study. There is not sufficient evidence to clearly state whether nutrients contributed to change the metabolisms activated by the inoculated fungi, or if by triggering the autochthonous microbiota, competition phenomena weakened their activity. It has been suggested that these readily metabolized sugars might have triggered the activation of nutritionally deprived autochthonous organisms and their competitiveness with the bioaugmented strains (Potin et al. 2004).

The chemical characterization of the soil, alone, does not allow us to accurately assess the risk associated. This is mainly because bioremediation may change pollutants bioavailability and create transformation intermediates, whose toxicity is largely undetermined (Shen et al. 2016). Ecotoxicological studies could instead evaluate the effectiveness of the treatments, considering also all the possible synergic effects caused by numerous and various compounds. Among the ecotoxicological tools used for soil risk assessment, *Eisenia foetida* is widely used, thanks to its exceptional ecological characteristics, sensitivity to contaminants and ease of use under controlled conditions (Kokta 1992; OECD 1984). Indeed, *E. foetida* is known to react to heavy metals (Neuhauser et al. 1985), microplastics (Ding et al. 2021), antibiotics (Zhou et al. 2024) and pesticides (Wang et al. 2021). Mortality was not negligible in the untreated soil, but there were no detectable toxic effects after performing the treatments (both BA, BS and BA-BS). As regards the second-generation endpoint, treatments did not change the toxicity of the soil with the only exception of BS. These data underline how the approach of not-doing-anything in a polluted area is not a smart solution. Even a pro-active BS system (with organic material to stimulate the autochthonous mycobiota) can produce significant benefits to the soil quality. On the other hand, the data demonstrated that the toxicity did not increase, making this phenomenon a risky backfire for many bio-based approaches. Indeed, the pollutant concentration decrease should not be wrongly attributed to a complete mineralization of the compounds. Bioremediation processes can instead lead to the formation of intermediate products that are more toxic than their parent compounds. Those products can therefore accumulate, and the toxicity of soil could result unaltered or even increased (Shen et al. 2016). Soroldoni and collaborators (2019) tested the soil toxicity after bioaugmentation and biostimulation treatments; despite the 98% reduction of PAHs in the soil, the system’s toxicity increased. In the present study, the reduction in toxicity in treatments with FW indicates that the soil improver most likely played a key role in reducing soil toxicity. On the other hand, BA treatment did not lead to any significant change in comparison with the beginning despite causing the highest PAHs removal. Treated soil is indeed a heterogeneous matrix with parent compounds as well as secondary metabolites produced by the fungal catabolic metabolism. The overall toxicity includes the effects of each compound and the possible synergic effects they can trigger. Unfortunately, it is difficult to outline which compound and/or intermediate could be responsible for the measured toxicity due to the scarcity of data reported on this topic. Surely *E. foetida* can be affected by secondary metabolites: phenolic hydrolytic products of parathion, an insecticide. were even more toxic than the parent material (Roberts and Wyman Dorough 1984). To lower further the toxicity of the soil, probably the elimination of degradation secondary products through a more prolonged bioremediation treatment could be useful, also in association with the use of soil improver. Targeted assessment of PAHs degradation products will shed light on the metabolic pathways activated by fungi, the mineralization rate and the potential residual toxicity after treatment.

## Conclusions

In this study, bioaugmentation and biostimulation approaches were evaluated for the treatment of PAHs-contaminated soil. All the systems were efficient, even though bioaugmentation led to higher contaminants transformation including the more recalcitrant HMW PAHs. Further studies should prioritize the optimization of the formulation of the fungal inoculum to enhance its colonization and availability in the soil, as it is known to be a crucial factor for the success of in situ clean-up of soils destined to urban agriculture. Moreover, the metabolic pathway expressed by the microbial community as well as the characterization of the degradation products are fundamental steps to a better understanding of the fate of PAHs in soil.

The success of this approach was led possibly by the initial choice to investigate the microbial community naturally populating this contaminated ecological niche. This study identified many species that have not been reported previously in the literature as occurring in PAHs-contaminated soil, capable of degrading them or producing biosurfactants. This novel biodiversity would surely represent a biological richness for future applications.

## Supplementary Information

Below is the link to the electronic supplementary material.

ESM 1 (DOCX 43.2 KB)

## Data Availability

The authors declare that the data supporting the findings of this study are available within the paper and its Supplementary Information files. Should any raw data files be needed in another format they are available from the corresponding author upon reasonable request.
