# Supplementary material for: Restoration of urban agriculture soil by autochthonous fungal biodiversity
Source: Appl Microbiol Biotechnol. 2026 May 28;110(1):226. doi: 10.1007/s00253-026-13877-z (PMC13407478; doi:10.1007/s00253-026-13877-z)
Supplement: Supplementary file 1 — (DOCX 43.2 KB) [file 253_2026_13877_MOESM1_ESM.docx]

Restoration of urban agriculture soil by autochthonous fungal biodiversity.

SUPPLEMENTARY MATERIAL

Matteo Florio Furno^1^, Michela Tramontini^2^, Elisa Gaggero^2^, Monica Rigoletto^2^, Debora Fabbri^2^, Mery Malandrino^2^, Paola Calza^2^, Giovanna Cristina Varese^1^, Federica Spina^1^

^1^University of Torino, Dept. of Life Sciences and Systems Biology, Viale Pier Andrea Mattioli 25, 10125 Torino, Italy

^2^University of Torino, Dept. of Chemistry, Viale Pietro Giuria 7, 10125 Torino, Italy

Corresponding authors:

Giovanna Cristina Varese

[cristina.varese@unito.it](mailto:cristina.varese@unito.it)

Federica Spina

federica.spina@unito.it

**Table S1** Compost Florawiva® characterization

| **Parameter** | **Value*** |
| --- | --- |
| origin | degradation and maturation of green and organic waste from separate waste collection |
| pH | 7-8.8 |
| Organic nitrogen | 20% (w/w) |
| C/N ratio | 25 |
| Humic carbon (humic acids + fulvic acids) | 7% |
| Electrical conductivity | 1.8 mS/cm |

*values declared by the producer - <https://ambiente.aceapinerolese.it/>

**Table S2** US EPA sixteen PAH priority pollutants with their chromatographic retention time and ions.

| **Compound** | **Retention time (min)** | **Molecular ion (m/z)** | **SIM ions (m/z)*** |
| --- | --- | --- | --- |
| Naphtalene | 5.8 | 128 | **128** |
| Acenaphthylene | 8.3 | 152 | **152**, 151 |
| Acenaphtene | 8.6 | 153 | **154**, 153, 152 |
| Fluorene | 9.4 | 165 | **166**, 165 |
| Phenantrene | 10.9 | 178 | **178** |
| Anthracene | 11.0 | 178 | **178** |
| Antrhracene D-10 | 10.9 | 188 | **188** |
| Fluoranthene | 12.8 | 202 | **202,** 200 |
| Pyrene | 13.2 | 202 | **202** |
| Benzo(a)anthracene | 15.1 | 228 | **228,** 226, 229 |
| Chrysene | 15.2 | 228 | **228**, 226 |
| Benzo(b)fluoranthene | 16.7 | 252 | **252** |
| Benzo(k)fluoranthene | 16.8 | 252 | **252,** 126 |
| Benzo(a)pyrene | 17.2 | 252 | **252** |
| Indeno(1,2,3-c,d)pyrene | 18.6 | 276 | **276,** 278, 279 |
| Dibenzo(a,h)anthracene | 18.6 | 278 | **278** |
| Benzo(g,h,i)perylene | 18.9 | 278 | **276,** 277, 274 |

*bold *m/z* are quantifier ions; other *m/z* are qualifier ions

**Table S3** Limit of Detection (LOD) and Limit of Quantification (LOQ) of PAHs

| PAH | LOD (μg/kg) | LOQ  (μg/kg) | PAH | LOD  (μg/kg) | LOQ  (μg/kg) |
| --- | --- | --- | --- | --- | --- |
| Naphtalene | 1.5 | 4.9 | **Crysene** | 2.9 | 9.7 |
| Acenaphtylene | 1.8 | 5.9 | **Benzo(b)fluoranthene** | 3.55 | 11.8 |
| Acenaphtene | 1.5 | 4.9 | **Benzo(k)fluoranthene** | 3.0 | 10 |
| Fluorene | 1.6 | 5.3 | **Benzo(a)pyrene** | 4.2 | 14 |
| Phenantrene | 0.8 | 2.7 | **Indeno(1,2,3-C,D)pyrene** | 3.78 | 12.6 |
| Anthracene | 1.2 | 4.1 | **Dibenzo(a,h)anthracene** | 2.03 | 6.8 |
| Fluoranthene | 1.8 | 5.9 | **Benzo(g,h,i)perylene** | 1.8 | 5.9 |
| Pyrene | 1.7 | 5.8 | **Anthracene D10** | 1.0 | 3.3 |
| Benzo(a)anthracene | 3.2 | 10.7 |  |  |  |

**Table S4** PAHs concentration of the urban agriculture site

| PAHs | Concentration  (μg/kg ± Dev. Std) | limits for  green areas  (μg/kg) | limits for agricultural areas (μg/kg) | limits for industrial areas (μg/kg) |
| --- | --- | --- | --- | --- |
| Naphtalene | 23.2 ± 6.7 | - | - | - |
| Acenaphthylene | 11.1 ± 4.5 | - | - | - |
| Acenaphtene | 25.2 ± 2.2 | - | - | - |
| Fluorene | 23.0 ± 1.8 | - | - | - |
| Phenantrene | 468.3 ± 54.9 | - | - | - |
| Anthracene | 116.8 ± 11.5 | - | - | - |
| Fluoranthene | 884.4 ± 73.2 | - | - | - |
| Pyrene | 717.2 ± 45.1 | 5,000 | - | 50,000 |
| Benzo(a)anthracene | 487.9 ± 88.6 | 500 | 1,000 | 1,0000 |
| Chrysene | 735.1 ± 110.3 | 5,000 | 1,000 | 5,0000 |
| Benzo(b)fluoranthene | 1161.7 ± 154.5 | 500 | 1,000 | 1,0000 |
| Benzo(k)fluoranthene | 745.9 ± 135.4 | 500 | 1000 | 1,0000 |
| Benzo(a)pyrene | 691.5 ± 91.6 | 100 | 100 | 1,0000 |
| Indeno(1,2,3-C, D)pyrene | 633.5 ± 20.7 | 100 | 1,000 | 50,000 |
| Dibenzo(a,h)anthracene | 463.0 ± 120.9 | 100 | 100 | 10,000 |
| Benzo(g,h,i)perylene | 634.5 ±75.0 | 100 | 5,000 | 10,000 |

**Table S5** Mesocosm (500 kg each) experiment setup

| **Mesocosm experiment** | **Fungal consortium (kg)** | **Organic amendment (kg)** |
| --- | --- | --- |
| Natural attenuation (C) | / | / |
| Biostimulation (BS) | / | 50 |
| Bioaugmentation (BA) | 50 | / |
| Biostimulation and Bioaumention (BS-BA) | 50 | 50 |

**Table S6** Average recovery obtained from the internal standard signal for each type of sample

| **Soil sample type** | **Average recovery** |
| --- | --- |
| Natural attenuation - C | 77.1% |
| Biostimulated - BS | 108.2 % |
| Bioaugmented soil - BA | 83.3% |
| Bioaugmented and biostimulated soil – BA-BS | 101.5 % |

**Table S7** Fungal taxa isolated from different substrates: paraffin oil (Pa), phenanthrene (Phe), pyrene (Pyr), crude oil (Oi), perylene (Pe) and eicosane (Ei). Fungal taxa are listed in alphabetical order.

| Taxa | Pa | Ph | Py | Oi | Pe | Ei |
| --- | --- | --- | --- | --- | --- | --- |
| *Aspergillus fumigatus* | x |  |  | x | x | x |
| *Aspergillus nidulans* |  |  |  | x | x |  |
| *Aspergillus niveus* |  |  |  |  | x |  |
| *Aspergillus terreus* | x |  |  | x | x | x |
| *Cladosporium pseudocladosporioides* |  |  |  | x |  |  |
| *Clonostachys rosea* | x |  |  |  |  |  |
| *Fusarium oxisporum* |  |  |  | x |  |  |
| *Fusarium proliferatum* |  |  | x |  |  |  |
| *Fusarium solani* |  |  |  |  |  | x |
| *Galactomyces psedocandidum* |  |  |  | x |  | x |
| *Hyphodermella rosae* |  |  |  |  |  | x |
| *Lecythophora canina* |  |  |  |  | x |  |
| *Metarhizium robertsii* |  |  |  | x |  |  |
| *Neurospora sitophila* |  |  |  |  | x |  |
| *Penicillium camponoti* |  |  |  |  |  | x |
| *Penicillium sp.1* |  |  |  |  |  | x |
| *Penicillium sp.2* |  |  |  |  |  | x |
| *Pichia manshurica* |  |  | x |  |  |  |
| *Purpureocillium lilacinum* |  |  |  | x | x | x |
| *Scedosporium apiospermum* |  |  |  | x | x | x |
| *Scedosporium aurantiacum* |  |  |  | x |  |  |
| *Scedosporium dehogii* |  |  | x | x | x |  |
| *Scedosporium sp.1* | x |  |  |  |  |  |
| *Talaromyces trachyspermus* |  | x |  | x |  |  |
| *Termoascus crustaceus* |  |  |  |  | x |  |
| *Trichoderma asperellum* |  | x |  |  | x |  |
| *Trichoderma* *gamsii* |  |  |  | x |  |  |
| *Trichoderma hamatum* |  |  |  | x |  |  |

**Figure S8** PAHs concentrations determined at the end of the treatment in the 4 different conditions (C, BS, BA, BA-BS). Data are expressed as average [µg/kg] ± std. dev.

| **Compound** | **C** | **BS** | **BA** | **BA-BS** |
| --- | --- | --- | --- | --- |
| Naphtalene | 245.1 ± 9.3 | 249.2 ± 14.1 | 260.3 ± 36.7 | 228.4 ± 12.7 |
| Acenaphthylene | 8.0 ± 0.2 | 6.2 ± 0.8 | 5.5 ± 0.9 | 4.6 ± 0.7 |
| Acenaphtene | 38.7 ± 6.5 | 9.3 ± 1.4 | 6.5 ± 0.2 | 16.5 ± 1.7 |
| Fluorene | 41.1 ± 3.0 | 17.3 ± 2.3 | 18.5 ± 3.2 | 18.4 ± 1.7 |
| Phenantrene | 514.2 ± 35.4 | 237.7 ± 3.4 | 184.5 ± 8.5 | 304.7 ± 89.8 |
| Anthracene | 152.3 ± 7.5 | 60.4 ± 4.1 | 43.5 ± 3.4 | 77.7 ± 23.8 |
| Fluoranthene | 845.7 ± 49.3 | 483.2 ± 36.1 | 405.0 ± 6.4 | 562.0 ± 151.1 |
| Pyrene | 734.6 ± 14.0 | 359.4 ± 39.0 | 303.3 ± 5.8 | 432.6 ± 127.6 |
| Benzo(a)anthracene | 390.9 ± 0.6 | 312.8 ± 34.2 | 238.8 ± 30.8 | 287.2 ± 1.2 |
| Chrysene | 449.1 ± 7.6 | 379.5 ± 21.6 | 278.9 ± 2.1 | 314.7 ± 10.6 |
| Benzo(b)fluoranthene | 400.6 ± 51.7 | 404.6 ± 10.7 | 249.0 ± 48.7 | 224.3 ± 6.2 |
| Benzo(k)fluoranthene | 349.1 ± 30.5 | 291.2 ± 24.2 | 247.1 ± 26.7 | 257.6 ± 3.4 |
| Benzo(a)pyrene | 363.4 ± 4.5 | 274.1 ± 32.8 | 191.3 ± 19.1 | 181.0 ± 42.7 |
| Indeno(1,2,3-c,d)pyrene | 304.8 ± 9.0 | 261.8 ± 30.7 | 207.8 ± 24.3 | 224.7 ± 10.9 |
| Dibenzo(a,h)anthracene | 80.7 ± 6.1 | 76.9 ± 8.1 | 56.4 ± 6.0 | 58.0 ± 0.9 |
| Benzo(g,h,i)perylene | 304.1 ± 1.2 | 233.0 ± 23.9 | 203.8 ± 11.4 | 205.6 ± 13.0 |

**Figure S9** PAHs degradation percentage in all the treatments.

| **Compound** | **BS** | **BA** | **BA-BS** |
| --- | --- | --- | --- |
| Naphtalene | -6% | -2% | 7% |
| Acenaphthylene | 31% | 23% | 42% |
| Acenaphtene | 83% | 76% | 57% |
| Fluorene | 55% | 58% | 55% |
| Phenantrene | 64% | 54% | 41% |
| Anthracene | 71% | 60% | 49% |
| Fluoranthene | 52% | 43% | 34% |
| Pyrene | 59% | 51% | 41% |
| Benzo(a)anthracene | 39% | 20% | 27% |
| Chrysene | 38% | 16% | 30% |
| Benzo(b)fluoranthene | 38% | -1% | 44% |
| Benzo(k)fluoranthene | 29% | 17% | 26% |
| Benzo(a)pyrene | 47% | 25% | 50% |
| Indeno(1,2,3-c,d)pyrene | 32% | 14% | 26% |
| Dibenzo(a,h)anthracene | 30% | 5% | 28% |
| Benzo(g,h,i)perylene | 33% | 23% | 32% |
